# Neuropsychological Characteristics of Children with Mixed Autism and ADHD

**DOI:** 10.1155/2017/5781781

**Published:** 2017-07-25

**Authors:** Costanza Colombi, Mohammad Ghaziuddin

**Affiliations:** University of Michigan, Ann Arbor, MI, USA

## Abstract

Clinical heterogeneity is a well-established characteristic of autism spectrum disorder (ASD). While the comorbidity of ASD and ADHD is well known in clinical practice, relatively little research has examined the neuropsychological profile of children with ASD + ADHD. Our study showed significant differences in the neuropsychological characteristics of children with ASD + ADHD compared to those with ASD only. Children with ASD + ADHD showed higher symptoms of anxiety, worse working memory, and less empathy, as measured by the “Reading the Mind in the Eyes.” This suggests that having ADHD brings further challenges to individuals with ASD and may negatively impact their management and outcome. Our findings may have implications for clinical assessment as well as for intervention.

## 1. Introduction

Clinical heterogeneity is a well-established characteristic of autism spectrum disorder (ASD). In addition to the core features of social communication impairment and restricted/repetitive behaviors and interests, a large number of children with ASD present with the symptoms of hyperactivity, inattention, and impulsivity, which may be severe enough to merit an additional diagnosis of attention deficit hyperactivity disorder (ADHD) [1]. Although the DSM-IV-TR [2] did not allow for a diagnosis of ADHD in individuals with ASD, reports about the comorbidity between the two conditions started appearing soon after its publication [3]. Recent studies have confirmed the initial reports. Goldstein and Schwebach [4] found that 16 (59%) of 37 individuals with ASD met DSM-IV criteria for a diagnosis of ADHD. Ogino et al. [5] reported the presence of ADHD in 6 (37.5%) of a sample of 16 individuals with ASD. Keen and Ward [6] found comorbidity of ASD and ADHD in 27 (13.7%) of a sample of 196 individuals. Siminoff et al. (2008) in a community-based study conducted in the UK found that approximately a third of participants with ASD presented with additional ADHD. Miodovnik et al. [7] in an epidemiological study involving 1496 children with ASD found that 20% of the participants had received a diagnosis of ADHD prior to the diagnosis of ASD. Fewer studies have examined the occurrence of autistic symptoms in persons with ADHD because most studies of ADHD tend to exclude subjects with autism and/or intellectual disability. While exact figures are difficult to estimate, a large number of children with ADHD also present with symptoms of ASD [8, 9]. In addition to the comorbidity between the two conditions, reports have also suggested a possible overlap in their symptoms [7].

It is crucial to identify the comorbidity between ASD and ADHD because of its treatment implications. For example, compared with children with ADHD only, those with ADHD and ASD symptoms have a lower adaptive functioning and a poorer quality of life [10, 11]. In addition, children with ADHD and ASD symptoms, compared with those with ADHD only, tend to be prescribed more medications [12] and respond less to stimulants [13] and may respond better to alternative agents such as atomoxetine (Harterftkamp et al., 2014).

However, despite the group differences reported in the literature, it can be extremely difficult to differentiate between ASD and ADHD in individual cases. Historical data and presentation of symptoms may not be helpful. For example, social deficits occur in both the disorders. Although the social deficits of autism are typically described as being “reciprocal” in nature resulting from an inborn lack of “affective contact” [14] and those of ADHD are said to be the result of inattention and distractibility, the distinction is not always easy to make in clinical practice. Rating scales and observation schedules also are of limited value especially when hyperactivity and impulsivity are marked.

In such cases, a careful evaluation of the neuropsychological characteristics may be helpful in identifying the comorbidity of ASD and ADHD. Cognitive profiles of individuals with ASD and those with ADHD are well described. For example, in a rigorous study of high functioning individuals with ASD, Wilson et al. [15] found that, compared to typically developing individuals, subjects with autism showed deficits in social cognition, executive function, and motor performance. On the other hand, individuals with ADHD consistently showed deficits in their working memory (Merkt et al., 2014), while facial emotion and mental states recognition was impaired in persons with ASD. Thus, individuals with ASD consistently perform worse than control subjects on the “Reading the Mind in the Eyes” Test (Eyes Test) (Golan and Baron-Cohen, 2006; Lombardo et al., 2007; Baron-Cohen et al., 2011), which is an advanced task to assess Theory of Mind, an index of facial emotion recognition and mental states attribution. While the Eyes Test has not been used in ADHD, many accounts report difficulties in emotion recognition in individuals with ADHD [16, 17]. Some authors have suggested that affect processing impairments in ADHD are due to difficulties in emotion recognition rather than to deficits in attention and inhibition [18].

Limited research has been done on the neuropsychological characteristics of individuals with ASD + ADHD. Some authors have suggested that those with ASD + ADHD show an “additive profile” rather than a qualitatively different set of difficulties compared with those with ASD only (Antshel et al., 2015). For example, Andersen et al. [19] found that children with ASD + ADHD were more impaired on verbal working memory than children with ASD only and those with ADHD only. In an investigation of attention and inhibition, Tye and others [20] found that ASD + ADHD children had impairments in all domains evaluated, while ASD children presented difficulties in response preparation and conflict monitoring only and ADHD children presented abnormalities in omission errors and reaction time. The aim of our study was to extend the work on the neuropsychological profile of individuals with ASD + ADHD and to explore the hypothesis that subjects with ASD + ADHD show higher degrees of impairment in social cognition than those with ASD only.

## 2. Methods

### 2.1. Participants

The study was conducted at the Department of Psychiatry, University of Michigan, and approved by the Institutional Review Board (HUM00008815). The participants, drawn from referrals to the ASD Clinic, consisted of 22 children with ASD and 25 children with mixed ASD and ADHD. The demographics are presented in Table 1.

The diagnosis of ASD, based on the DSM-IV [2], was reached after a comprehensive evaluation by a multidisciplinary team consisting, among others, of a board-certified child/adolescent psychiatrist, a neuropsychologist, a speech pathologist, a social worker, and a fellow in child psychiatry. Both parents/caregivers and the participant were directly interviewed. In addition to the clinical interview, supplemental information was obtained from the case-notes, medical records, and school reports. Additional diagnostic information was obtained on the basis of the Autism Diagnostic Interview-Revised (ADI-R, [21]). The subtypes of DSM-IV PDD were collapsed together to form a single category. For the diagnosis of additional ADHD, the participant had to meet the DSM-IV criteria of ADHD [2]. As in the diagnosis of ASD, the subtypes of DSM-IV ADHD were collapsed into a single category. From both the groups, participants with known genetic conditions and/or marked sensory deficits were excluded. All children had to have a Full Scale IQ of at least 70 to be eligible to participate.

### 2.2. Procedure

Neuropsychological measures were administrated using standardized procedures. Measures, based on the main ASD deficits identified in the scientific literature, evaluated cognitive level of functioning, emotion recognition, Theory of Mind, executive functions, and motor abilities. The following measures were administered.

*The Wechsler Intelligence Scale for Children, 4th Edition (WISC IV)*. It includes 10 subtests, yielding four index scores that combine into the Full Scale IQ (FSIQ). The Verbal Comprehension Index (VCI) consists of three subtests (Similarities, Comprehension, and Vocabulary). The Perceptual Reasoning Index (PRI) comprises Block Design, Matrix Reasoning, and Picture Concepts. The Working Memory Index (WMI) comprises Digit Span and Letter-Number Sequencing. The Processing Speed Index (PSI) comprises Coding and Symbol Search. Index standard scores have a mean of 100 and a standard deviation of 15.

*Trail Making Test*. The Trail Making Test (TMT) [22] is a visuomotor task used to measure sustained visual attention and psychomotor speed. TMT part A requires the participant to rapidly draw lines to connect consecutively numbered circles on one worksheet. TMT part B requires connecting the same number of consecutively numbered and lettered circles by alternating between the two sets, which involves frequent switching between two mental sets.

*Tower of London*. The Tower of London (TOL; Shallice 1982) measures planning and problem solving performance. The mechanical version of the TOL used in this study consists of two identical wooden game boards, one for the participants and one for the examiner. Each game board includes three different size pegs and three different colored balls. The participant needs to move the balls on the pegs to match the examiner's game board in the minimum number of moves.

*The Strange Stories Task [23]*. It consists of vignettes depicting social situations. The task requires the participant to explain a character's behavior with reference to his or her mental states. This is a measure of Theory of Mind.

*Reading the Mind in the Eyes, Revised Version [24]*. The revised version of the Eyes Test [24] consists of 36 black-and-white photographs of the eye region. Each trial requires the participant to choose among four descriptors related to the feelings of the person in the picture. This test is considered a measure of Theory of Mind.

*The Child Behavior Checklist (CBCL) [25]*. It is a measure assessing disruptive behavior and social competency in children. Caregivers of the participants completed the Child Behavior Checklist (CBCL). We used the CBCL to confirm ADHD symptoms.

### 2.3. Statistical Analysis

Statistical analyses were performed using the IBM SPSS Statistics software (Version 23). We first performed independent samples *t*-tests comparing the two groups in terms of age and IQ. We then performed univariate analyses of covariance (ANCOVAs), comparing the two groups while including IQ as a continuous covariate, for the following dependent variables: working memory, Children's Color Trail Test, Tower of London, Category Fluency Test, Happe's Stories, and Reading the Mind in the Eyes Test.

## 3. Results

Descriptive statistics for the two groups are reported in Table 1. Means and standard deviations for the experimental measures are reported in Table 2. The independent samples *t*-tests showed that the two groups did not differ in terms of mean age, mean Full Scale IQ, mean Verbal IQ, and mean Performance IQ. ADHD symptoms (*t* = −2.19; *p* = 0.03) and anxiety symptoms (*t* = −1.95; *p* = 0.05), as measured by the CBCL, were higher in the group of children with ASD + ADHD compared with the ASD only group. Based on the results of the ANCOVAs, there was a significant difference between the two groups in working memory (*F*(1,34) = 4.52; *p* = 0.041), with the ASD only group having a significantly higher mean when holding FIQ fixed, indicating that children with ASD + ADHD present more impairment in working memory. Additionally, there was a marginally significant difference in the Reading the Mind in the Eyes Test (*F*(1,44) = 3.44, *p* = 0.07), with the ASD only group having a marginally higher mean when controlling for FIQ, indicating that the ASD + ADHD group present slightly higher difficulties in the Eyes Test. The Trail Making Test, Tower of London, Category Fluency Test, and Happe's Stories did not show significant differences between the groups.

## 4. Discussion

In 1902, about 50 years before Leo Kanner described autism, George Still laid down the main characteristics of the condition that later came to be known as ADHD (see Goldstein, 2006). While Kanner did not describe the comorbid disorders his eleven patients suffered from, a close reading of his seminal paper suggests that at least a few of them seemed to show symptoms that would today be diagnosed as ADHD. Before the DSM-5 [26] was published, it was believed that the two diagnoses of ASD and ADHD were mutually exclusive. The diagnosis of ADHD could not be given in persons with ASD on the assumption that symptoms of inattention and hyperactivity were rather common in those with autism resulting in artefactual comorbidity. However, research in the last two decades has established that despite the seeming overlap between the symptoms of the two disorders and the challenges of diagnosing ADHD in some patients with ASD, such as those with severe intellectual disability (mental retardation), there does seem to exist a subgroup of patients who have both the disorders based on behavioral features. The results of this study provide preliminary neuropsychological evidence of the existence of this patient group.

The main finding of this study is that compared with children who have ASD only those who have ASD + ADHD are more impaired in their ability to read other people's emotions and feelings (“Reading the Mind in the Eyes Test”) and to hold and manipulate critical periods of information (“working memory”). Thus, when we compared the neuropsychological profiles of 22 children with ASD only with those of 25 children with ASD + ADHD, we found significant differences between the two groups. Working memory was significantly more impaired in children with additional ADHD. Moreover, a marginally significant difference was identified in the Eyes Test, indicating that children with ASD + ADHD may have more difficulties in emotion recognition. Regarding behavioral symptoms, children with ASD + ADHD showed higher levels of anxiety in line with other studies [19, 20]; however, we did not identify qualitative differences in the profiles of the two groups. The worse performance in working memory and in emotion recognition, along with the higher levels of anxiety found in individuals with ASD + ADHD, supports the hypothesis that having additional ADHD increases the level of their impairment and negatively impacts their management and outcome.

It is to be noted that the sample consisted only of those with normal level of intelligence. Therefore, the findings may not apply to lower functioning children with ASD and ADHD. The risk of intellectual disability (ID) increases the risk of both ASD and ADHD, acting alone and in combination with other factors. Indeed, diagnosing the two conditions in those with severe and profound intellectual disability is extremely difficult. Since working memory is impaired in those with ID [27], it can be speculated that lower functioning children with ASD and ADHD are more impaired in measures of social cognition than those with normal intelligence. Future studies should examine the effect of decreasing IQ on ASD children with comorbid ADHD. Other limitations of the study also need to be considered when interpreting our results. Thus, it is possible that the performance was affected by tiredness in our participants due to the large test battery administered. Finally, the size of our sample may have failed to identify differences.

In summary, identifying symptoms of ADHD in children with ASD has important diagnostic and treatment implications. However, a reliable diagnosis based only on developmental history and clinical observation is not always possible. Examining the cognitive and neuropsychological deficits in children with both the conditions can facilitate their diagnosis and inform treatment. Our results also confirm the greater overall impairment in children with ASD plus ADHD as indexed by their performance on tests of social cognition. Further studies in subjects with a wider range of cognitive abilities, using larger sample sizes of different age groups, should be undertaken to better define the profiles of individuals with ASD + ADHD and identify effective pharmacological and behavioral interventions.

## Figures and Tables

**Table 1:** Background information.

Variable	ASD	ASD + ADHD	*F*	*p*
	Mean (SD)	Mean (ASD)		
Age	10.9 (3.6)	9.8 (2.2)	6.08	0.21
FIQ	91.5 (18.8)	90.8 (15.6)	0.56	0.89
VIQ	96.2 (21.6)	95.7 (15.4)	2.21	0.92
PIQ	94.2 (18.7)	97.2 (16.6)	1.23	0.56

**Table 2:** Means and standard deviations for the experimental measures.

Variable	ASD	ASD + ADHD	*F*	*p*
	Mean (SD)	Mean (SD)		
Working memory	93.00 (13.66)	85.78 (13.09)	4.5	0.04
Trail A	40.00 (18.43)	42.68 (14.27)	0.80	0.37
Trail B	46.75 (20.76)	45.22 (11.54)	0.15	0.69
TOL	51.20 (18.25)	52.30 (9.63)	0.37	0.54
Categories	51.23 (11.86)	48.70 (10.51)	0.55	0.46
Stories	5.86 (4.07)	5.39 (3.66)	0.09	0.75
Eyes	15.95 (3.28)	14.00 (3.98)	3.43	0.07
Anxiety (CBCL)	59.87 (10.21)	65.57 (7.49)	3.74	0.05
Withdrawal (CBCL)	62.81 (10.28)	65.71 (9.93)	−0.86	0.39
Attention (CBCL)	64.50 (11.20)	72.09 (9.78)	−2.19	0.03
Social (CBCL)	63.75 (12.10)	67.42 (9.26)	−1.04	0.30
